# An Integrated QSM‐Radiomics Nomogram With Clinical and Imaging Markers for Stratifying Cognitive Impairment in Hypertension

**DOI:** 10.1002/cns.70769

**Published:** 2026-01-30

**Authors:** Yu Su, Tingting Liu, Chengjun Dong, Limin Ge, Tianxiang Li, Zhiqing Zhang, Yihan Zhang, Chungao Li, Jie Zhao, Chuansheng Zheng, E. Mark Haacke, Wenjun Wu, Lixia Wang

**Affiliations:** ^1^ Department of Radiology, Union Hospital, Tongji Medical College Huazhong University of Science and Technology Wuhan China; ^2^ Hubei Provincial Clinical Research Center for Precision Radiology & Interventional Medicine Wuhan China; ^3^ Hubei Key Laboratory of Molecular Imaging Wuhan China; ^4^ SpinTech MRI Bingham Farms Michigan USA; ^5^ Department of Radiology Wayne State University Detroit Michigan USA

**Keywords:** cognitive impairment, hypertension, iron, quantitative susceptibility mapping, radiomics

## Abstract

**Background:**

Hypertensive cognitive impairment is associated with increased iron deposition in deep gray matter. This study aimed to evaluate the potential clinical application of quantitative susceptibility mapping (QSM)‐based radiomics for stratifying cognitive impairment in hypertensive patients.

**Methods:**

We prospectively enrolled 178 hypertensive patients who underwent QSM examination and categorized them into cognitive impairment group and normal cognition group. The workflow included: (1) Precise 3D segmentation and susceptibility quantification of the basal ganglia; (2) Initial radiomics feature extraction from the target regions, followed by optimal feature selection via variance thresholding, maximum relevance minimum redundancy (mRMR) algorithm, and LASSO regression; (3) Construction of a multiparametric model integrating radiomics scores and clinical risk factors. The model's performance was assessed by receiver operating characteristic (ROC) curve and its clinical utility for stratification was further evaluated using decision curve analysis (DCA).

**Results:**

The multi‐region radiomics model demonstrated superior diagnostic performance in the training cohort (AUC = 0.812), significantly outperforming the QSM and WMH models (AUCs = 0.620 and 0.688, respectively). In the validation cohort, clinically meaningful improvements were observed (ΔAUC = 0.117 and 0.136, respectively). The combined model, which integrated Radscore, susceptibility values, WMH scores, age, and education level, achieved the highest discriminability in both the training (AUC = 0.860) and validation cohorts (AUC = 0.872). DCA further indicated that the nomogram derived from the combined model provided the greatest net clinical benefit for stratifying hypertensive cognitive impairment.

**Conclusion:**

A nomogram integrating QSM‐based radiomics with clinical and imaging markers accurately stratified hypertensive cognitive impairment, offering an objective tool for early risk assessment.

## Graphical abstract

This study developed an integrated nomogram combining QSM‐based radiomics, brain iron deposition, white matter hyperintensity, and clinical markers. This model demonstrated superior performance in stratifying hypertensive cognitive impairment compared to traditional single‐parameter models. It offers a valuable non‐invasive tool for early risk assessment and personalized intervention.

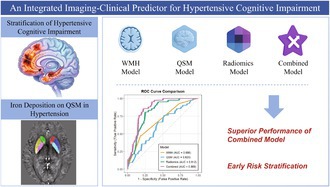

## 1 Introduction

Hypertension (HTN) is a major vascular risk factor worldwide, significantly increasing the risk of cerebrovascular events and serving as a key contributor to vascular cognitive impairment and dementia [1]. The association between HTN and cognitive decline has been well‐established [2, 3], underscoring the importance of early identification for timely intervention and delaying dementia progression. HTN can induce and exacerbate cognitive impairment, making early detection crucial for preventing and delaying the progression of dementia. However, current clinical practice still lacks effective tools for accurately stratifying patients at risk or in the early stages of the disease. Widely used neuropsychological scales, such as the Montreal Cognitive Assessment (MoCA) and the Mini‐Mental State Examination (MMSE), are reliable and valid but are often too complex for routine clinical screening [4]. Additionally, hypertensive cognitive impairment (CI) presents with subtle early symptoms and slow pathological progression, and there remains a lack of objective biomarkers for early detection.

HTN induces pathological alterations in cerebral microvasculature, leading to structural, connectivity, and functional impairments. These microvascular injuries manifest as characteristic neuroimaging markers on MRI, including cerebral microbleeds, lacunar infarcts, and white matter hyperintensities (WMH) [5]. Beyond these vascular dysfunction‐related markers, dysregulated iron metabolism and its deposition in the brain have gained increasing attention in CI. A large‐scale cross‐sectional study demonstrated that iron metabolism markers, including serum iron and ferritin levels, are significantly associated with HTN incidence [6]. Furthermore, evidence suggests elevated iron deposition in specific deep gray matter in hypertensive patients [7, 8, 9]. Conventional MRI sequences (T1WI, T2WI, FLAIR) are capable of identifying lacunar infarcts or white matter lesions; however, they exhibit limited sensitivity in detecting early and subtle iron metabolism abnormalities in deep gray matter.

In recent years, quantitative susceptibility mapping (QSM), an emerging MRI technique, enables noninvasive, high‐resolution quantification of tissue iron content with exceptional sensitivity to susceptibility values [10]. Moreover, studies have indicated that HTN‐related vascular iron leakage and hypoxia‐induced iron dysregulation contribute to excessive cerebral iron deposition, which is closely associated with cognitive decline [7, 8]. Gray matter nuclei (particularly the basal ganglia), with higher vascular density and perfusion demands [11], are more vulnerable to HTN‐induced injury. However, susceptibility values alone reflect global iron levels but may not capture microstructural heterogeneity. Radiomics, by extracting high‐dimensional subvisual features from medical images, offers a powerful tool for quantifying tissue heterogeneity [12]. Previous studies have demonstrated that QSM‐based radiomics effectively diagnose Parkinson's disease (PD) and are associated with CI [13]. A recent study supported the worth of combining QSM and radiomics for early cerebral small vessel disease (CSVD) diagnosis [14]. However, no study has yet investigated the application of radiomics for CI stratification in patients with HTN.

This study aimed to investigate whether radiomics based on QSM can effectively stratify hypertensive CI. By integrating high‐dimensional radiomic features, regional iron deposition, WMH burden, and clinical risk factors, we sought to construct and validate a multiparameter combined diagnostic model to enhance stratification accuracy. Furthermore, we planned to develop a visualized nomogram based on the optimal model to facilitate early identification of high‐risk individuals with subclinical CI. The outcomes of this research are anticipated to provide a decision‐making tool to support screening, early objective identification, and personalized intervention strategies for hypertensive CI.

## 2 Materials and Methods

### 2.1 Participants and Grouping

This study was approved by the Ethics Committee of Union Hospital, Tongji Medical College, Huazhong University of Science and Technology (Ethics Approval No. UHCT21811) and strictly adhered to the principles of the Declaration of Helsinki. From September 2019 to December 2024, 232 hypertensive patients were prospectively enrolled, with 178 ultimately meeting the inclusion criteria. Inclusion criteria were: confirmed HTN for ≥ 1 year with regular antihypertensive medication use; exclusion criteria included secondary HTN, severe parenchymal brain lesions, other systemic malignancies, or incomplete imaging data. Blood pressure was measured three times using a portable monitor, and the mean was recorded. HTN was defined as resting systolic blood pressure (SBP) ≥ 140 mmHg and/or diastolic blood pressure (DBP) ≥ 90 mmHg [15].

**FIGURE 1:**
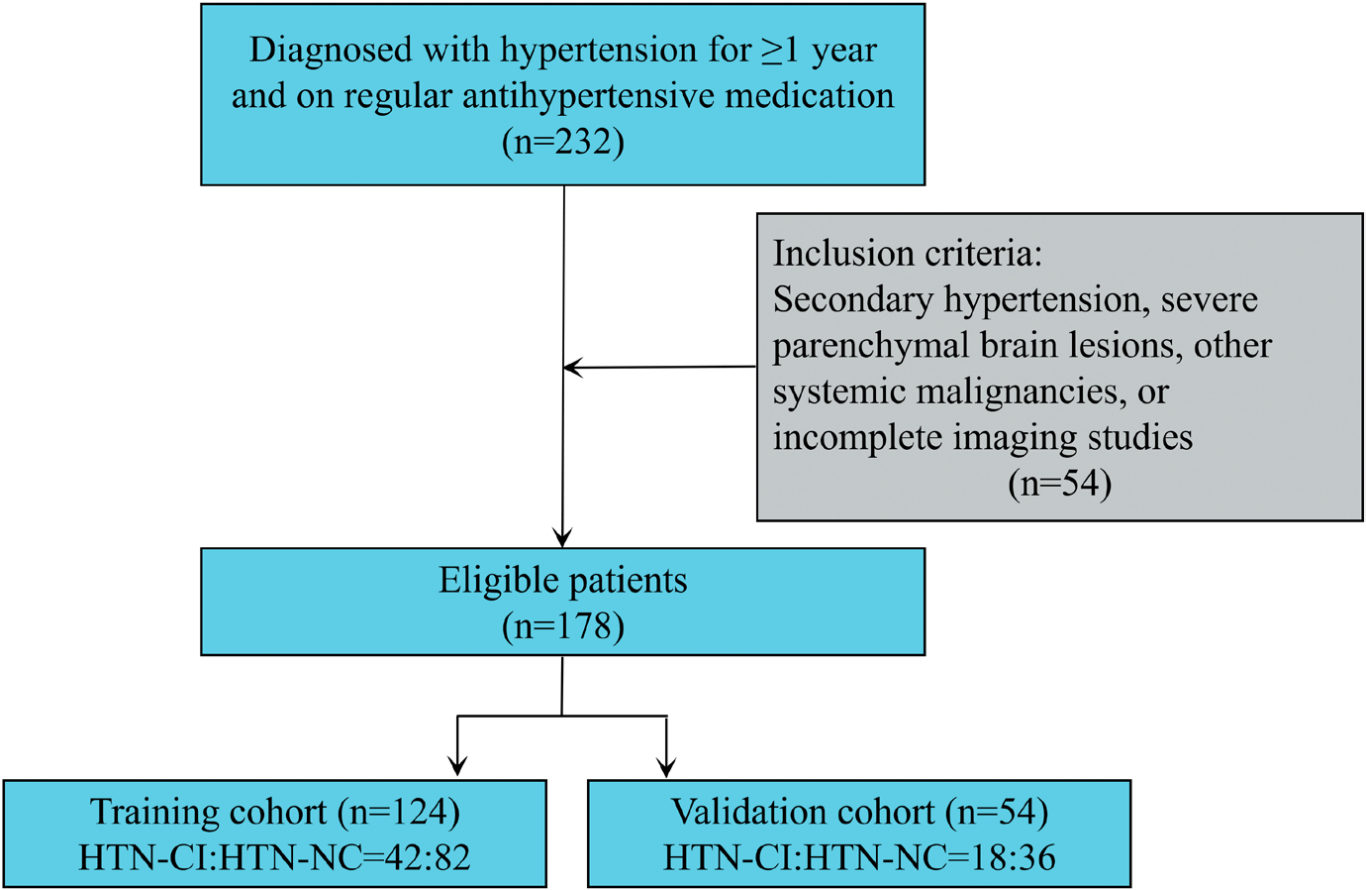
Flowchart of subject enrollment and exclusion criteria, as well as allocation to the training cohort and validation cohort.

Cognitive function was assessed using the Beijing version of the MoCA, which evaluates seven domains (visuospatial/executive function, naming, delayed recall, attention, language, abstraction, and orientation) with a total score of 30. Education‐adjusted cutoff values were applied: ≤ 13/14 for illiterate individuals, ≤ 19/20 for primary education, and ≤ 24/25 for junior high school or higher [16]. Based on MoCA scores, patients were classified into HTN with cognitive impairment (HTN‐CI) and HTN with normal cognition (HTN‐NC) groups. All assessments were independently completed by participants within 10 min. Finally, patients were randomly divided into a training cohort (*n* = 124, 70%) and a validation cohort (*n* = 54, 30%) (Figure 1).

### 2.2 MRI Data Acquisition

All participants underwent brain MRI on a 3.0‐T Siemens Skyra scanner (Siemens Healthcare, Erlangen, Germany) using a 32‐channel head coil. The Strategic Acquisition Gradient Echo (STAGE) sequence was employed, performing two fully flow compensated double‐echo gradient echo acquisitions within 336 s [17, 18]: (i) Axial dual‐echo proton density‐weighted (PDW) imaging (repetition time [TR] = 25 ms, echo time [TE] = 7.5/17.5 ms, flip angle = 6°) (ii) Axial dual‐echo T1‐weighted (T1W) imaging (TR = 25 ms, TE = 8.75/18.75 ms, flip angle = 24°). Shared parameters included field of view (FOV) = 256 × 256 mm^2^, matrix = 384 × 144, voxel size = 0.67 × 1.33 × 2.0 mm^3^, slice thickness = 2 mm, and 64 slices. Additional axial FLAIR, axial T1W, and sagittal T2W images were acquired for anatomical reference and lesion assessment.

### 2.3 MRI Postprocessing and Measurements

Medical image preprocessing and QSM reconstruction: Raw MRI data were preprocessed using SPIN software (SpinTech MRI, Bingham Farms, MI, USA) to establish the STAGE pipeline. QSM reconstruction was performed using STAGE software (v2.1.4) based on PDW and T1W images, involving [19]: (i) Skull stripping and phase unwrapping (ii) Background field removal (iii) QSM reconstruction via iterative susceptibility‐weighted imaging. Three experienced neuroradiologists (C.D., Y.S., and L.G.) manually delineated regions of interest (ROIs) using ITK‐SNAP (v4.0.2, http://www.itksnap.org) while carefully excluding vessels and adjacent ventricular structures. Bilateral caudate nucleus head (CN), putamen (PU), and globus pallidus (GP) were segmented, and susceptibility values (in parts per billion, ppb) were averaged across raters. For the grading of WMH, two senior neuroradiologists (C.L. and W.W., with 11 and 8 years of experience in neuroradiology, respectively) independently evaluated the periventricular and deep WMH on FLAIR images using the Fazekas scale [20] under blinded conditions. The total scores (range 0–6) were calculated as the sum of both regional scores. Any discrepancies were adjudicated by a third chief radiologist (L.W., with 16 years of experience).

### 2.4 Radiomics Feature Extraction and Selection

This study employed a standardized pipeline for radiomics feature extraction and selection. Based on QSM imaging data, the PyRadiomics open‐source software package (https://pyradiomics.readthedocs.io/en/latest/) was used to extract a set of radiomic features from each gray matter nucleus that showed statistically significant intergroup differences in susceptibility values. The extracted features per nucleus included first‐order statistics (18 features), shape‐based features (14 features), gray‐level co‐occurrence matrix (GLCM) features (24 features), gray‐level run‐length matrix (GLRLM) features (16 features), gray‐level size zone matrix (GLSZM) features (16 features), neighboring gray‐tone difference matrix (NGTDM) features (5 features), and gray‐level dependence matrix (GLDM) features (14 features). A total of 107 features were extracted from each original image. Additionally, multimodal filtering was applied to the original images using 13 types of filters (including wavelet transform, LoG filtering, square transform, etc.) [21]. From these filtered images, six categories of radiomic features were extracted, with shape features computed only on the original images. Ultimately, a high‐dimensional dataset comprising 85 feature categories and a total of 1130 features was constructed. Each feature was uniquely identified using the naming convention: “image type–feature group–feature name”.

We used t‐SNE (perplexity = 30, learning rate = 200) to reduce the high‐dimensional feature space (1130 dimensions) to two dimensions while preserving the data structure based on Euclidean distance. The results demonstrated consistent distributions between the training and validation cohorts.

**FIGURE 2:**
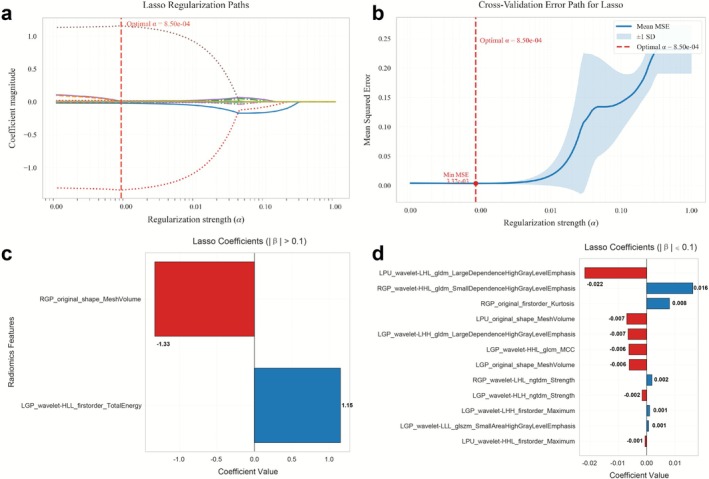
Distribution of LASSO coefficients for radiomic features. (a) LASSO coefficient profile of 19 radiomic features. A vertical line was drawn at the α value determined by 10‐fold cross‐validation, where the optimal α value corresponded to 14 radiomic features. The final optimal α value was selected as 0.00085. (b) The red vertical line indicates the value selected via 10‐fold cross‐validation in (a). (c, d) Histograms of radscore ((c) Features with absolute LASSO coefficients > 0.1, (d) Features with absolute LASSO coefficients < 0.1). The *y*‐axis represents the 14 selected radiomic features, and the *x*‐axis denotes their coefficient values.

A multi‐stage feature reduction strategy was applied exclusively to the training cohort to prevent data leakage into the validation cohort: initially applying variance thresholding (threshold = 0.01) to eliminate low‐variance features, followed by Z‐score normalization to standardize the training cohort features. Subsequently, the minimum redundancy maximum relevance (mRMR) algorithm was employed to identify the top 1–30 cognition‐relevant features, which were then further refined through LASSO regression with 10‐fold cross‐validation (using the 1‐SE criterion to determine α, Figure 2a,b) to ultimately select the 14 most optimal features (Figure 2c,d) that demonstrated the strongest association with cognitive outcomes.

### 2.5 Model Construction, Radiomics Nomogram, and Performance Evaluation

**FIGURE 3:**
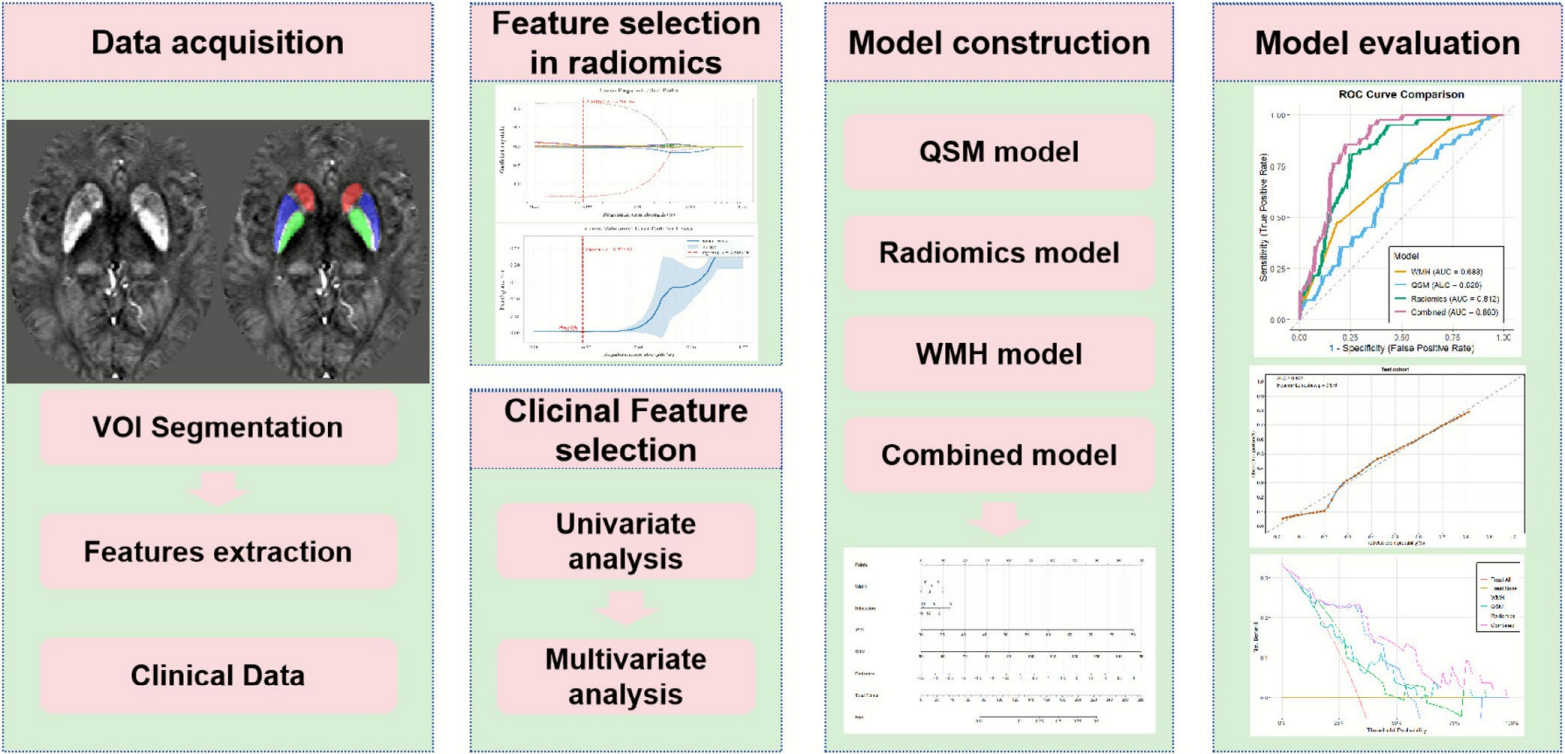
The flowchart of this study.

We developed a radiomics score (radscore) derived from the selected features and constructed four distinct predictive models: a QSM model based on susceptibility values, a radiomics model using Radscore, a WMH model, and a comprehensive combined model incorporating QSM, Radscore, WMH, and clinical factors. Through univariate logistic regression, we firstly identified significant predictors, which were subsequently analyzed using multivariate regression for the combined model. The diagnostic performance of all models was rigorously evaluated using receiver operating characteristic (ROC) curves to determine sensitivity, specificity, accuracy, positive predictive values (PPV), and negative predictive values (NPV). To enhance clinical applicability, we created a nomogram that visually represents the combined model's predictive capabilities, complemented by calibration curves and decision curve analysis (DCA) to thoroughly assess its reliability and clinical utility. The complete workflow was shown in Figure 3.

### 2.6 Statistical Analysis

Analyses were performed using Python (v3.12.7) and R (v4.5.1). Continuous variables were reported as mean ± standard deviation or median (interquartile range); categorical variables as frequency (%). Group differences were tested via independent samples *t*‐test, Mann–Whitney *U* test, *χ* ^2^ test, or Fisher's exact test. Intraclass correlation coefficient (ICC) was assessed for susceptibility measurements. Feature selection employed variance thresholding, mRMR, and LASSO. Model AUCs were compared via DeLong test. The nomogram was constructed using the R “rms” package and internally validated via bootstrap resampling (1000 iterations). Calibration was tested with Hosmer–Lemeshow. All tests were two‐tailed, with *p* < 0.05 considered significant.

## 3 Results

### 3.1 Clinical Characteristics

A total of 178 patients (74 males, 104 females; 60 in the HTN‐CI group, 118 in the HTN‐NC group) with a mean age of 58.99 ± 9.94 years were included. Table 1 summarizes the baseline characteristics. Compared to the validation cohort, the training cohort had a higher proportion of smoking (*p* = 0.028), yet no significant differences were observed in other baseline characteristics. Importantly, there were also no significant statistical differences in the proportion of CI between the training and validation cohorts, indicating the balanced division of the dataset (Table 1).

**TABLE 1:** Baseline clinical and imaging characteristics of the hypertensive patients.

Characteristics	Training cohort (*n* = 124)	Validaiton cohort (*n* = 54)	*p*
Year^a^	59.05 ± 10.08	58.85 ± 9.69	0.904
Gender^b^	78 (62.9%)	26 (48.1%)	0.066
Education^c^	9 (6, 12)	9 (7, 15)	0.467
Smoking^b^	46 (37.1%)	11 (20.4%)	0.028*
Drinking^b^	41 (33.1%)	21 (38.9%)	0.453
SBP^a^	138 (127, 146)	141 (129, 150)	0.195
DBP^a^	84 (77, 92)	86 (80, 93)	0.233
Hypertension duration^c^	8 (3, 13)	6 (3, 12)	0.939
Diabetes^b^	29 (23.4%)	13 (24.1%)	0.923
Hyperlipidemia^b^	38 (30.6%)	21 (38.9%)	0.283
Periventricular WMH^c^	1.27 ± 0.65	1.31 ± 0.58	0.638
Deep WMH^c^	1.00 ± 0.59	1.00 ± 0.64	0.967
WMH^c^	2.27 ± 1.09	2.31 ± 1.00	0.797
MOCA^c^	23 (18, 26)	23 (18, 26)	0.398
Cognition^b^			
HTN‐CI	42 (33.9%)	18 (33.3%)	0.944
HTN‐NC	82 (66.1%)	36 (66.7%)	

^a^ *p*‐value was obtained through independent samples *t*‐test.

^b^ *p*‐value was obtained through appropriate χ^2^ test or Fisher's exact test.

^c^ *p*‐value was obtained through the Mann–Whitney *U*‐test.

* The *p*‐value < 0.05.

### 3.2 Comparison of Susceptibility Values

Inter‐rater reliability for susceptibility measurements across different gray matter nuclei, assessed via ICC, exceeded 0.920 for all three radiologists (Table S1), indicating excellent reproducibility. The study results indicate that there were no statistically significant differences in susceptibility values of gray matter nuclei between the training cohort and the validation cohort (*p* > 0.05) (Table 2). Further subgroup analysis based on cognitive function revealed that, compared to the HTN‐NC, the HTN‐CI showed significantly higher susceptibility values in the bilateral GP and left PU regions (*p* < 0.05), whereas there were no statistically significant differences in susceptibility values of other gray matter nuclei (Table S2).

**TABLE 2:** Comparison of susceptibility values (ppb) between training cohort and validation cohort.

ROIs	Training cohort (*n* = 124)	Validaiton cohort (*n* = 54)	*p*
Right RCN^a^	39.52 ± 16.29	39.02 ± 17.83	0.854
Left LCN^a^	39.98 ± 23.36	36.93 ± 11.42	0.363
Right RGP^a^	114.45 ± 31.25	108.33 ± 27.92	0.217
Left LGP^a^	114.53 ± 32.09	108.46 ± 31.39	0.244
Right RPU^a^	56.44 ± 23.36	55.95 ± 21.77	0.896
Left LPU^a^	57.42 ± 21.23	61.05 ± 21.90	0.300

^a^ *p*‐value was obtained through independent samples *t*‐test. The *p*‐value < 0.05 was considered statistically significant.

### 3.3 Feature Selection and Radscore Construction

This study selected the bilateral GP and Left PU, which exhibited significant inter‐group differences, as feature regions, extracting a total of 3390 radiomic features for subsequent analysis. The t‐SNE visualization results of the training and validation cohorts showed no significant differences in feature distribution, indicating a reasonable data split. After variance thresholding (threshold = 0.01), 2329 features were eliminated, retaining 1061. Subsequent mRMR and LASSO regression selected 14 non‐zero‐coefficient features from the multi‐region fusion set (Figure 3). The corresponding Radscore was derived as a weighted linear combination of these selected features; the complete formula was presented in the Data S1. For comparative purposes, radscores were also constructed for the RGP, LGP, and LPU based on their respective selected features.

### 3.4 Performance Comparison of Radiomics, QSM, WMH, Single‐Region, and Combined Models

**FIGURE 4:**
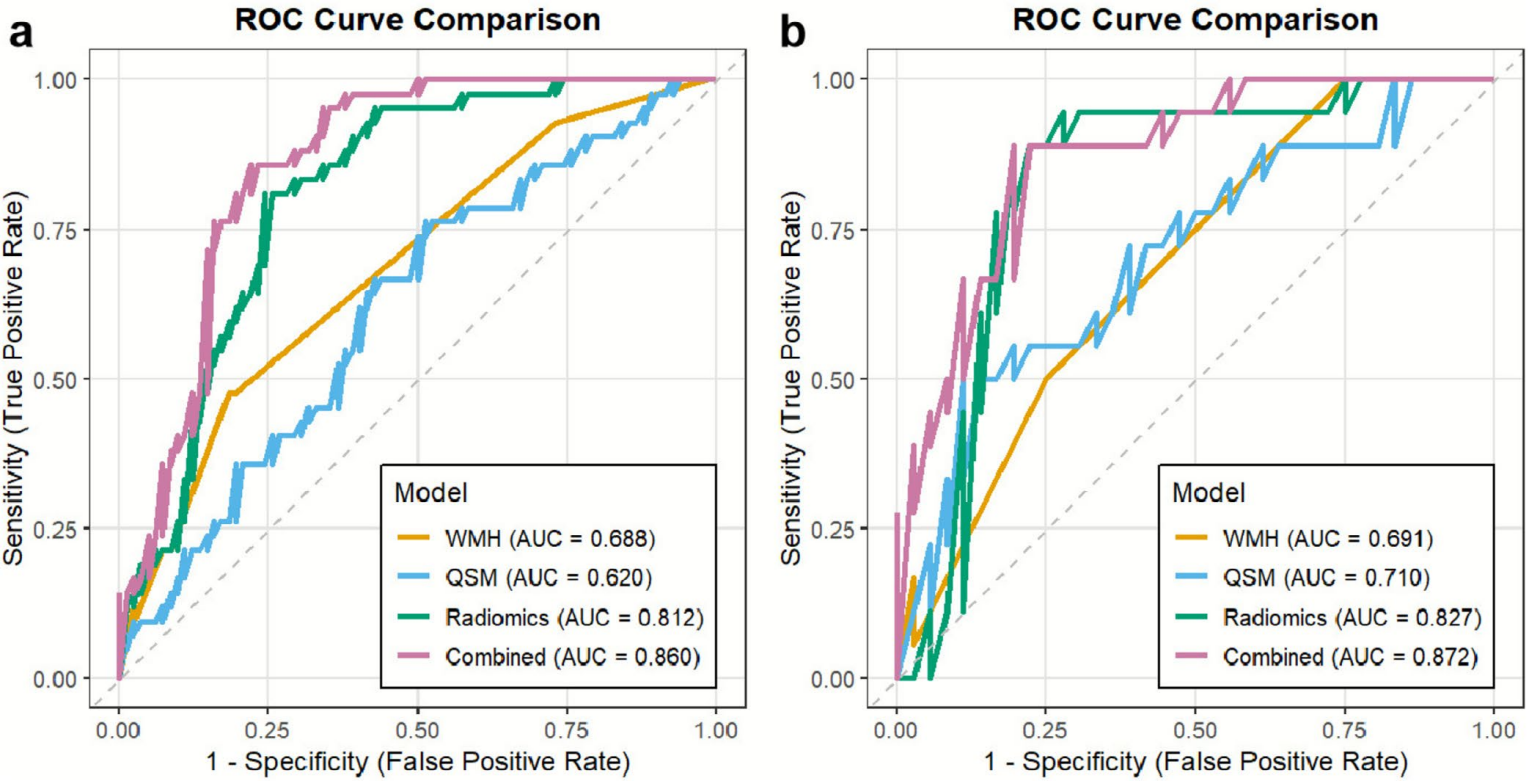
ROC curve comparison of QSM model, radiomic model, WMH model, and combined model for diagnosing HTN‐CI in the training cohort (a) and validation cohort (b).

The boxplot analysis of Radscore (Figure S1) revealed significant discriminatory power between HTN‐CI and HTN‐NC groups (*p* < 0.001, Wilcoxon test), with both univariate and multivariate regression analyses confirming Radscore as an independent predictor of CI. Through univariate analysis, we further identified mean susceptibility values of the three ROIs, age, education level, and WMH scores as potential predictors, which were subsequently integrated with Radscore to develop the combined model (Table 3). As shown in Table 4 and Figure 4, the multi‐region radiomics model demonstrated robust diagnostic performance, achieving AUC of 0.812 (95% CI: 0.742–0.902) in the training cohort and 0.827 (95% CI: 0.707–0.947) in the validation cohort. The DeLong test indicated that the radiomics model was significantly superior to the single QSM and WMH models in the training cohort (AUC = 0.812 vs. 0.620, *p* = 0.002; AUC = 0.812 vs. 0.688, *p* = 0.020). Although the validation cohort did not reach statistical significance, it showed a clinically meaningful improvement (ΔAUC = 0.117 and 0.136, *p* = 0.263 and 0.061, respectively). Furthermore, we constructed single‐region radiomics models based on the right GP (RGP‐radiomics), left GP (LGP‐radiomics), and left PU (LPU‐radiomics). Their performance was detailed in Figure S2. In the validation cohort, the multi‐region radiomics model (Radiomics) significantly outperformed both the RGP‐radiomics model (AUC = 0.827 vs. 0.610, *p* < 0.001) and the LGP‐radiomics model (AUC = 0.827 vs. 0.631, *p* = 0.001), and showed a strong trend toward superiority over the LPU‐radiomics model (AUC = 0.827 vs. 0.667, *p* = 0.070). Subsequently, we developed a multiparameter combined model incorporating the Radscore, susceptibility values, WMH scores, age, and education level. Although DeLong tests showed that the AUC improvement of the combined model over the Radiomics model was not statistically significant in either the training cohort (*p* = 0.065) or validation cohort (*p* = 0.393), consistent increases in AUC were observed (ΔAUC = 0.048 and 0.045, respectively).

**TABLE 3:** Univariate and multivariate logistic regression analyses of factors associated with hypertensive cognitive impairment.

Characteristics	Univariate analysis		Multivariate analysis (Combined model)	
	Odds Ratio (95% CI)	*p*	Odds Ratio (95% CI)	*p*
WMH	1.964 (1.329–2.903)	< 0.001*	1.410 (0.881–2.257)	0.152
Education	0.817 (0.739–0.903)	< 0.001*	0.874 (0.778–0.982)	0.024*
Year	1.057 (1.014–1.102)	0.009*	0.994 (0.938–1.053)	0.836
QSM	1.026 (1.004–1.048)	0.021*	1.022 (0.994–1.051)	0.124
Radscore	2.397 (1.669–3.444)	< 0.001*	2.168 (1.447–3.250)	< 0.001*

* The *p*‐value < 0.05.

**TABLE 4:** Comparison of diagnostic performance among QSM model, radiomic model, WMH model and combined model in the training cohort and validation cohort.

Model	AUC (95% CI)	Accuracy (%)	Sensitivity (%)	Specificity (%)	PPV (%)	NPV (%)
WMH model						
Training cohort	0.688 (0.598–0.778)	0.70	0.82	0.48	0.57	0.75
Validaiton cohort	0.691 (0.561–0.821)	0.67	0.50	0.75	0.50	0.75
QSM model						
Training cohort	0.620 (0.517–0.723)	0.58	0.76	0.49	0.43	0.80
Validaiton cohort	0.710 (0.559–0.861)	0.76	0.50	0.89	0.69	0.78
Radiomic model						
Training cohort	0.812 (0.742–0.902)	0.77	0.81	0.76	0.63	0.89
Validaiton cohort	0.827 (0.707–0.947)	0.83	0.89	0.81	0.70	0.94
Combined model						
Training cohort	0.860 (0.796–0.924)	0.81	0.86	0.78	0.67	0.91
Validaiton cohort	0.872 (0.776–0.968)	0.83	0.89	0.81	0.70	0.94

### 3.5 Development and Validation of the Nomogram

**FIGURE 5:**
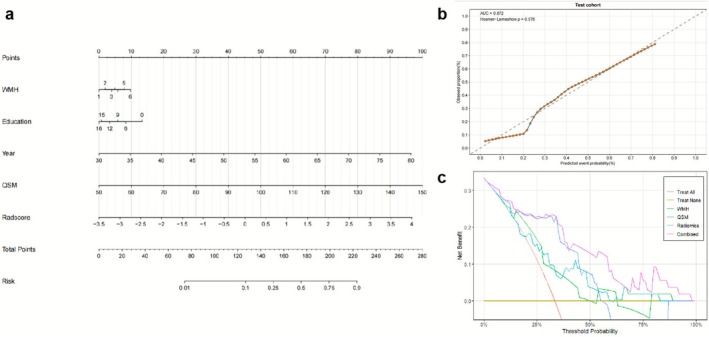
Nomogram model constructed based on risk factors identified through multivariate logistic regression analysis. (a) Combined nomogram for diagnosing hypertensive cognitive impairment. (b) Calibration curve showing agreement between predicted probabilities and actual incidence in the validation cohort. (c) Decision curve analysis comparing net benefits of six clinical scenarios for hypertensive cognitive impairment risk prediction:QSM model (blue line), Radiomic model (green line), WMH model (orange line), Combined model (pink line), All (assuming all patients have cognitive impairment), and None (assuming all patients have normal cognition).

**FIGURE 6:**
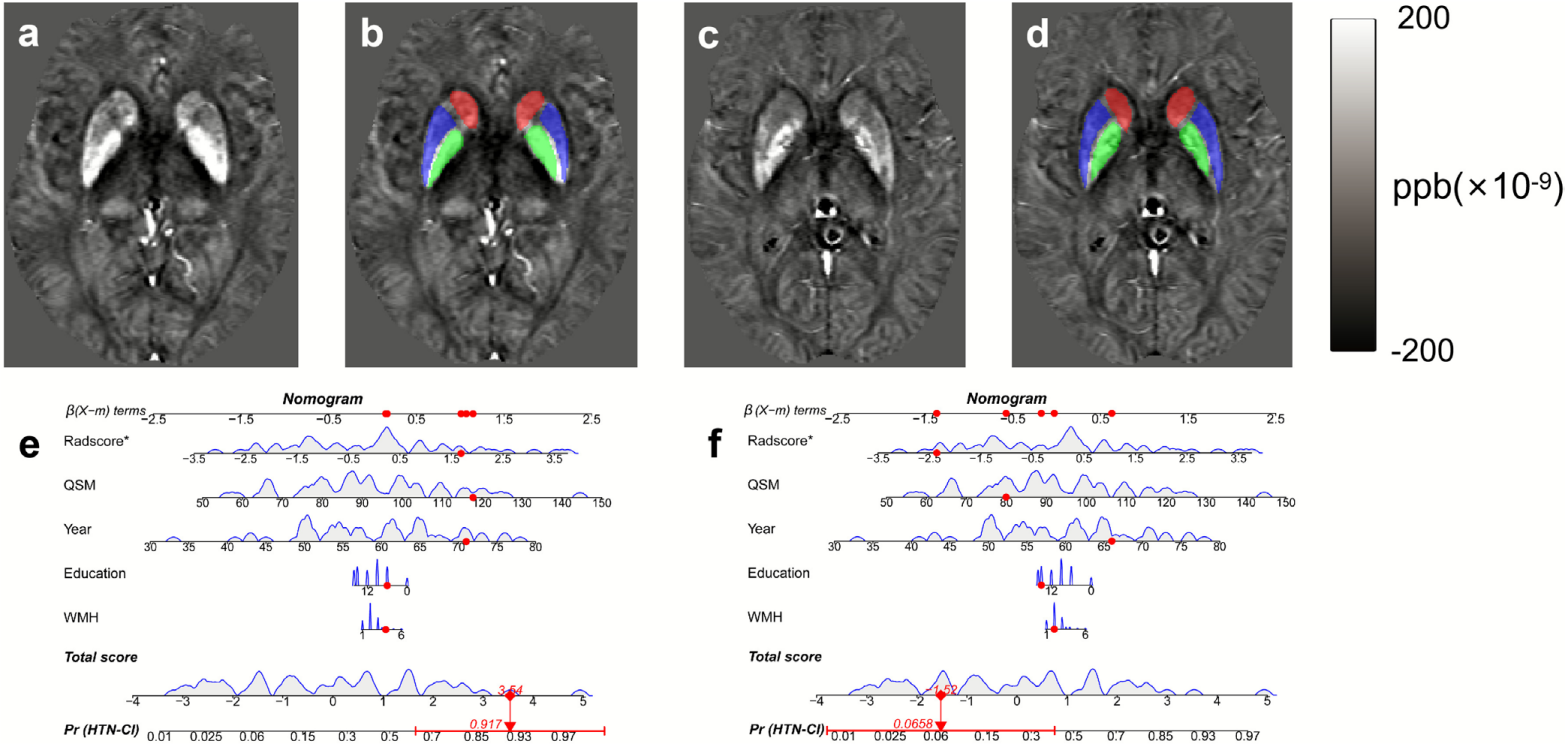
Clinical application of the nomogram for cognitive impairment risk in hypertensive patients. (a, b) A 71‐year‐old male (6 years of education) demonstrates diffuse iron deposition in basal ganglia on quantitative susceptibility mapping (QSM), WMH scores of 4, and radscore of 1.69, yielding a total nomogram score of 3.54 corresponding to 91.7% predicted cognitive impairment probability, which correlates well with the impaired Montreal Cognitive Assessment (MoCA) score of 13. (c, d) In contrast, a 66‐year‐old male (15 years of education) shows mild iron deposition, WMH scores of 2, and radscore of −2.36, resulting in a total score of −1.52 (6.6% cognitive impairment probability), consistent with preserved cognitive function (MoCA = 25).

The developed nomogram (Figure 5a) effectively integrates radiomics features and clinical predictors into a user‐friendly visual tool for individualized stratified assessment of hypertensive CI, demonstrating excellent calibration with Hosmer‐Lemeshow tests showing strong agreement between predicted and observed probabilities in both training cohorts (*p* = 0.184) and validation cohorts (*p* = 0.578) (Figure 5b). DCA (Figure 5c) results showed that the combined model demonstrated significant advantages in clinical applicability. Compared with the WMH model, the QSM model, and the radiomics model, the combined model had the widest range of clinical decision thresholds. When the clinical decision threshold exceeded 0.1, the combined model provided a more significant clinical net benefit in predicting hypertensive CI. As illustrated in Figure 6, clinicians can readily apply this tool by assigning points for each predictor (including WMH, age, education level, QSM, and Radscore), summing these to obtain total scores that correspond to a specific probability of hypertensive CI on the nomogram's risk scale, thereby enabling practical stratification of patients into high‐risk versus low‐risk categories to guide personalized intervention strategies.

## 4 Discussion

This study systematically explored the application value of radiomics features based on QSM images in the cognitive stratification of hypertensive patients and successfully constructed a multiparametric combined diagnostic model. The 14 radiomics features selected by machine learning algorithms from the basal ganglia exhibited excellent diagnostic efficacy, with the radiomics model significantly outperforming traditional QSM or WMH single‐parameter models. More importantly, we further integrated radscore, susceptibility values of bilateral GP and Left PU, Fazekas scores, and clinical risk factors to build a comprehensive model, which achieved an AUC value of 0.872 in the validation cohort. Based on this model, we further developed a clinically practical nomogram tool, whose intuitive visual scoring system provides clinicians with an objective and quantitative decision‐making basis.

In hypertensive CI, cerebral iron deposition was not only increased but also heterogeneously distributed, predominantly in the basal ganglia. QSM enables both qualitative and quantitative detection of iron deposition. In this study, significant differences in iron deposition were observed in the bilateral GP and left PU, consistent with previous findings [7, 8, 22]. Two recent studies on CSVD cohorts also found that iron deposition in the CN, PU, and GP was significantly associated with cognitive decline [23, 24]. Additionally, a study on hereditary small vessel disease reported increased iron deposition in the CN and PU of Cerebral Autosomal Dominant Arteriopathy with Subcortical Infarcts and Leukoencephalopathy (CADASIL) patients, with iron burden significantly correlated with cognitive deterioration [25]. These findings suggest that QSM‐based measurement of iron deposition in the basal ganglia may serve as an important diagnostic approach for CI in vascular diseases. However, the diagnostic efficacy of using QSM measurements alone to distinguish hypertensive CI remains unsatisfactory.

Radiomics features demonstrated unique value in assessing CI. In this study, the 14 selected radiomics features could be categorized into three types (shape features, first‐order features, and texture features), all extracted from wavelet‐filtered or original images. These features exhibited statistically significant differences between the HTN‐CI and HTN‐NC groups. Essentially, they reflect information on pixel intensity and texture distribution that is imperceptible to radiologists during manual assessment. Shape features describe the size and morphology of ROIs in three‐dimensional space, while first‐order features primarily reflect the distribution of voxel intensities within the imaging region. Texture features—including GLCM, GLSZM, NGTDM, and GLDM—characterize the spatial relationships of pixel gray values in the image. Wavelet features mainly capture texture and intensity distribution information across multiple scales and directions [26]. Notably, the right GP volume (RGP_original_shape_MeshVolume) and left GP wavelet energy (LGP_wavelet‐HLL_firstorder_TotalEnergy) contributed the most in distinguishing HTN‐CI and HTN‐NC, reflecting that the deep gray matter atrophy and non‐uniform iron aggregation, respectively, contribute to CI. Compared to conventional QSM, which provides only a single parameter (mean susceptibility value), radiomics enables multidimensional analysis of the spatial heterogeneity of iron deposition through high‐throughput feature extraction, aligning with the findings of Kang et al. [13] in PD research. Cheng et al. [14] reported that increased iron deposition in the GP of CSVD might be associated with cognitive decline, but diagnosis relying solely on QSM values lacks sufficient pathophysiological evidence. The introduction of radiomics features effectively addresses this limitation, offering new insights into the mechanisms underlying iron deposition‐related CI.

To validate the rationale for multi‐region feature fusion, we constructed single‐region radiomics models. The results demonstrated that the multi‐region fusion model achieved significantly superior diagnostic performance in the validation cohort compared with the single RGP model and the LGP model, and also showed a trend toward improvement over the single LPU. These findings support our hypothesis that hypertensive CI involves coordinated pathological alterations across multiple gray matter nuclei, and that integrating multi‐regional features can more comprehensively and sensitively capture the spatial heterogeneity of iron deposition, thereby providing better predictive performance than any single‐region model.

Moreover, integrating age, education level, and WMH Fazekas scores into the nomogram significantly improved model performance. DCA demonstrated greater clinical net benefit at threshold probabilities > 10%, confirming the reliability of the nomogram as a noninvasive screening tool. Univariate analysis revealed significant associations between hypertensive CI and age, WMH, education level, and susceptibility values, corroborating existing research on the pathological mechanisms linking vascular risk factors, education, iron deposition, and CI [27, 28, 29]. However, in the multivariate model, only education level and radscore emerged as independent predictors of CI. Education, as an early cognitive stimulus, is a key modifiable protective factor against dementia [28]. The crucial impact of educational level on cognitive reserve may have obscured the subtle effects of age, which aligns with the known sensitivity of the MoCA scale to education level [30]. Although susceptibility values lost their independent significance in the multivariate analysis, the persistent significance of radscore further underscores how radiomics, by decoding spatial heterogeneity patterns in iron deposition, provides pathological insights that cannot be achieved by traditional quantitative parameters. As the most prevalent MRI manifestation of CSVD, WMH significantly increase dementia risk and are closely associated with its pathological progression [21]. However, in our multivariate analysis, their independent predictive value was obscured and diagnostic performance remained limited. This may be attributable to our reliance on the Fazekas scale for basic visual assessment without further quantification of WMH volume or extraction of deeper imaging information [21]. Importantly, although DeLong tests showed that the AUC improvement of the combined model over the radiomics model did not reach statistical significance, the consistent upward trend in AUC and the highest overall discriminative performance support the clinical utility of integrating readily available clinical and imaging markers.

This study has several limitations. First, the modest sample size and single‐center recruitment may limit the generalizability of the model. Specifically, the small validation cohort likely contributed to insufficient statistical power, which may explain why the observed improvements of the radiomics model over the QSM and WMH models did not reach statistical significance. Second, while manual ROI segmentation enables high‐precision delineation of gray matter nuclei, it is considerably more time‐consuming; automated approaches may provide a more efficient alternative without compromising accuracy. Third, this study primarily focused on the basal ganglia and did not incorporate cortical gray matter structures. The STRIVE‐2 criteria highlight cortical iron deposition as a core imaging feature of CSVD [31], and animal studies have also shown that iron overload in the motor cortex can lead to motor neuron death [32]. Future studies should conduct multicenter validation with larger, independent cohorts to evaluate the model's robustness across different scanners and populations, and should also incorporate additional brain regions while exploring deep learning‐based automated segmentation techniques to optimize radiomics workflows.

## 5 Conclusion

This study successfully constructed a QSM‐based radiomics model, which demonstrated superior diagnostic performance in stratifying CI among hypertensive patients compared to conventional QSM and WMH models. By integrating multi‐region radiomic features, iron deposition quantification, WMH, and clinical risk factors, the developed multiparameter combined model further improved predictive stability. A visualized clinical nomogram was subsequently constructed based on this model. This tool provides a reliable imaging basis for the early and objective stratification of hypertensive CI, holding significant potential for clinical translation.

## Author Contributions

Yu Su and Tingting Liu: conceptualization, methodology, and writing – review and editing. Yu Su and Wenjun Wu: data curation, formal analysis, visualization. Chengjun Dong, Limin Ge and Tianxiang Li: resources, investigation. Zhiqing Zhang, Yihan Zhang and Chungao Li: investigation. Jie Zhao, E. Mark Haacke, and Chuansheng Zheng: software, writing – review and editing. Wenjun Wu and Lixia Wang: supervision and funding acquisition.

## Funding

The research was supported by the Hubei Provincial Key R&D Program (Grant number: 2022BCA034).

## Ethics Statement

This prospective observational investigation received ethical approval from the Institutional Review Board of Union Hospital, affiliated with Tongji Medical College at Huazhong University of Science and Technology (approval number: UHCT21811). Written informed consent was obtained from all participants prior to their inclusion. The research adhered to the principles outlined in the Declaration of Helsinki and all relevant revisions.

## Conflicts of Interest

The authors declare no conflicts of interest.

## Supporting information


**Table S1:** The ICC of susceptibility values in hypertensive patients assessed by three radiologists.
**Table S2:** Comparison of susceptibility values (ppb) between groups.
**Figure S1:** The Mann–Whitney *U* test revealed that the Radscore was significantly higher in the HTN‐CI group compared to the HTN‐NC group in both the training and test cohorts.***p < 0.001.
**Figure S2:** ROC curves comparing the performance of single‐region radiomics models (RGP‐radiomics, LGP‐radiomics, LPU‐radiomics) and the multi‐region fusion radiomics model (Radiomics) in diagnosing HTN‐CI in the training cohort (a) and the validation cohort (b).

## Data Availability

The datasets generated or analyzed during this study are available from the corresponding author upon reasonable request.
