# Supplementary material for: An Integrated QSM‐Radiomics Nomogram With Clinical and Imaging Markers for Stratifying Cognitive Impairment in Hypertension
Source: CNS Neurosci Ther. 2026 Jan 30;32(2):e70769. doi: 10.1002/cns.70769 (PMC12856517; doi:10.1002/cns.70769)
Supplement: Supplementary file 1 — Table S1: The ICC of susceptibility values in hypertensive patients assessed by three radiologists. Table S2: Comparison of susceptibility values (ppb) between groups. Figure S1: The Mann–Whitney U test revealed that the Radscore was significantly higher in the HTN‐CI group compared to the HTN‐NC group in both the training and test cohorts.***p < 0.001. Figure S2: ROC curves comparing the performance of single‐region radiomics models (RGP‐radiomics, LGP‐radiomics, LPU‐radiomics) and the multi‐region fusion radiomics model (Radiomics) in diagnosing HTN‐CI in the training cohort (a) and the validation cohort (b). [file CNS-32-e70769-s001.docx]

| **Table S1** The ICC of susceptibility values in hypertensive patients assessed by three radiologists | |
| --- | --- |
| ROIs | ICC (95% CI) |
| Right CN | 0.922 (0.901-0.939) |
| Left CN | 0.947 (0.932-0.959) |
| Right RGP | 0.975 (0.968-0.981) |
| Left LGP | 0.978(0.972-0.983) |
| Right RPU | 0.957 (0.945-0.967) |
| Left LPU | 0.951 (0.938-0.962) |

| **Table S2**Comparison of susceptibility values (ppb) between groups | | | |
| --- | --- | --- | --- |
| Characteristics | HTN-CI (n=60) | HTN-NC (n=118) | P value |
| Right RCN^a^ | 40.52 ± 15.74 | 38.79 ± 17.24 | 0.514 |
| Left LCN^a^ | 42.20 ± 28.73 | 37.46 ± 14.55 | 0.145 |
| Right RGP^a^ | 120.67 ± 31.50 | 108.48 ± 28.99 | 0.011* |
| Left LGP^a^ | 119.99 ± 35.50 | 108.98 ± 29.39 | 0.029* |
| Right RPU^a^ | 59.56 ± 21.63 | 54.628 ± 23.33 | 0.173 |
| Left LPU^a^ | 63.15 ± 21.13 | 56.17 ± 21.30 | 0.040* |
| ^a^P value was obtained through independent samples t-test. The p value < 0.05 was considered statistically significant. | | | |

**Radscore** = (-0.022 × LPU_wavelet-LHL_gldm_LargeDependenceHighGrayLevelEmphasis) + (0.001 × LGP_wavelet-LLL_glszm_SmallAreaHighGrayLevelEmphasis) + (0.002 × RGP_wavelet-LHL_ngtdm_Strength) + (-1.334 × RGP_original_shape_MeshVolume) + (0.016 × RGP_wavelet-HHL_gldm_SmallDependenceHighGrayLevelEmphasis) + (-0.006 × LGP_original_shape_MeshVolume) + (-0.007 × LGP_wavelet-LHH_gldm_LargeDependenceHighGrayLevelEmphasis) + (-0.001 × LPU_wavelet-HHL_firstorder_Maximum) + (0.001 × LGP_wavelet-LHH_firstorder_Maximum) + (0.008 × RGP_original_firstorder_Kurtosis) + (-0.006 × LGP_wavelet-HHL_glcm_MCC) + (-0.002 × LGP_wavelet-HLH_ngtdm_Strength) + (-0.007 × LPU_original_shape_MeshVolume) + (1.153 × LGP_wavelet-HLL_firstorder_TotalEnergy).


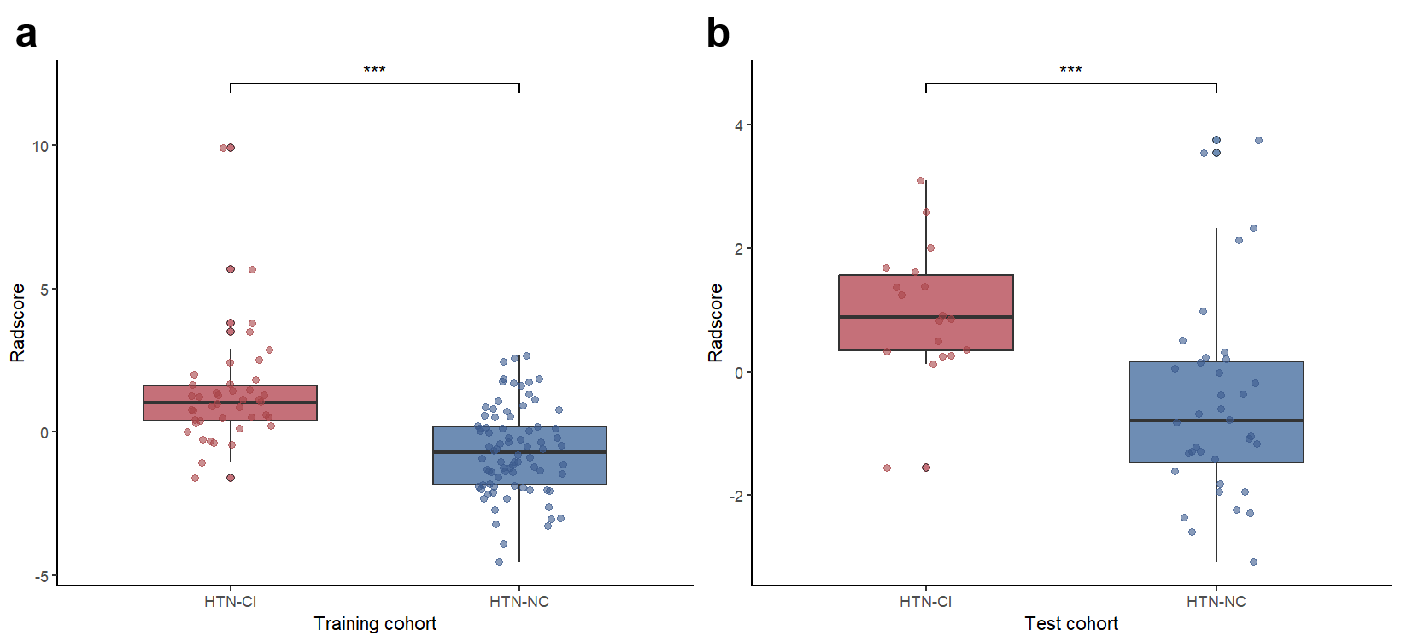


**Figure S1** The Mann-Whitney U test revealed that the Radscore was significantly higher in the HTN-CI group compared to the HTN-NC group in both the training and test cohorts.****P*＜0.001


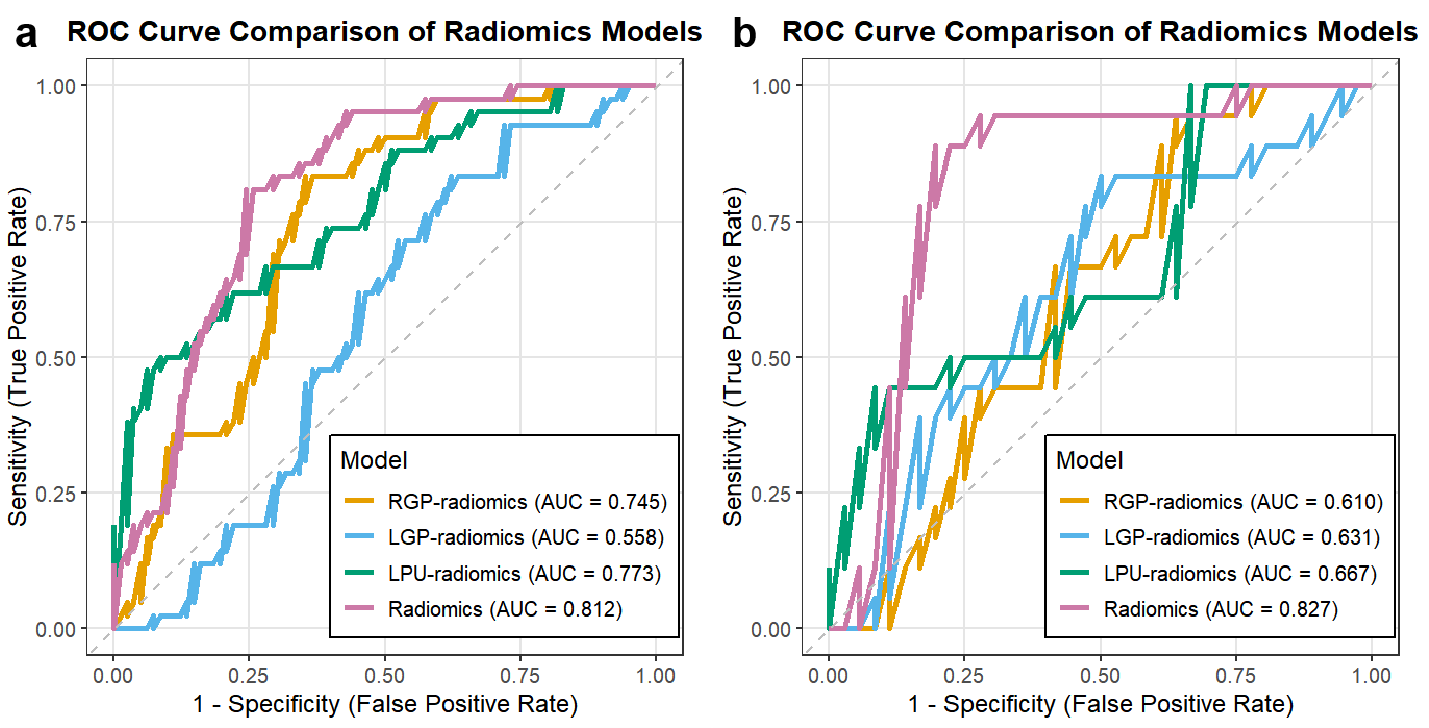


**Figure S2** ROC curves comparing the performance of single-region radiomics models (RGP-radiomics, LGP-radiomics, LPU-radiomics) and the multi-region fusion radiomics model (Radiomics) in diagnosing HTN-CI in the training cohort (a) and the validation cohort (b).
